# Mo_6_ cluster-based compounds for energy conversion applications: comparative study of photoluminescence and cathodoluminescence

**DOI:** 10.1080/14686996.2017.1338496

**Published:** 2017-07-03

**Authors:** Benjamin Dierre, Karine Costuas, Noée Dumait, Serge Paofai, Maria Amela-Cortes, Yann Molard, Fabien Grasset, Yujin Cho, Kohsei Takahashi, Naoki Ohashi, Tetsuo Uchikoshi, Stéphane Cordier

**Affiliations:** ^a^ Laboratory for Innovative Key Materials and Structures (LINK), UMI 3629 CNRS-Saint Gobain-NIMS, Tsukuba, Japan; ^b^ NIMS-Saint-Gobain Center of Excellence for Advanced Materials, National Institute of Material Science, Ibaraki, Japan; ^c^ Institut des Sciences Chimiques de Rennes (ISCR), UMR 6626 CNRS – University of Rennes 1, Rennes, France; ^d^ Research Center for Functional Materials, National Institute for Materials Science (NIMS), Tsukuba, Japan

**Keywords:** Energy conversion, metal clusters, molybdenum, ligands, counter-cations, photoluminescence, cathodoluminescence, molecular engineering, 40 Optical, magnetic and electronic device materials, 102 Porous / Nanoporous / Nanostructured materials, 204 Optics / Optical applications, 502 Electron spectroscopy

## Abstract

We report the photoluminescence (PL) and cathodoluminescence (CL) properties of face-capped [Mo_6_X^i^_8_L^a^_6_]^2−^ (X = Cl, Br, I; L = organic or inorganic ligands) cluster units. We show that the emission of Mo_6_ metal atom clusters depends not only on the nature of X and L ligands bound to the cluster and counter-cations, but also on the excitation source. Seven members of the A_x_Mo_6_X^i^_8_L^a^_6_ series (A = Cs^+^, (n-C_4_H_9_)_4_N^+^, NH_4_^+^) were selected to evaluate the influence of counter-cations and ligands on de-excitation mechanisms responsible for multicomponent emission of cluster units. This study evaluates the ageing of each member of the series, which is crucial for further energy conversion applications (photovoltaic, lighting, water splitting, etc.).

## Graphical abstract

**Figure:**
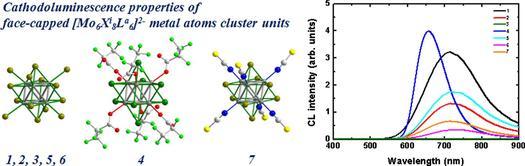

## 1. Introduction

Energy supply is one of the greatest challenges of humankind. The current world energy consumption of ca. 16 TW is expected to increase to more than 30 TW by 2050 [1]. Meanwhile, as CO_2_ emissions are predicted to increase (around 43 billion metric tons by 2035) there is an urgent need for alternatives to fossil energy sources. In this frame, solar energy appears as the most relevant alternative by taking advantage of a quasi-infinite and renewable resource. On the other side, buildings are one of the major sources of energy consumption; our society is clearly facing the challenges of green buildings. Thus, novel materials and processes are needed to meet these challenges, and composite nanomaterials could be one way to answer it [2]. One promising strategy is the development of new nanocomposite materials and concepts for smart windows and solar devices. The challenge to achieve this goal is to find less toxic and more efficient phosphors than the currently available materials based on rare earth (RE) or heavy metals (HM). Among the possible candidates, metal clusters (MCs), which consist of less than a few dozens of metal atoms, are receiving more and more attention owing to their attractive properties and great flexibility in terms of composition and design [3–6]. In the past decades, Mo_6_ metal cluster compounds have shown interesting and rich complexity of structural and physico-chemical properties [7,8]. Their good solubility in various solvents provides a wide range of processing routes to elaborate molecular assemblies and nanocomposite materials [6]. Thus, a large variety of Mo_6_-based MCs have been investigated for potential applications in lighting [6,9–11], solar cells [12–14], and biotechnologies [15–19]. Additionally, their high chemical flexibility has already allowed the fabrication of transparent nanocomposite thin films in organic or inorganic matrices that can be easily coated on substrates for photonic applications [20,21] Moreover, in addition to their very interesting optical properties for energy conversion applications (molecule-like energy gaps, strong photoluminescence in near infrared (NIR) region, etc.), they have also some specific electronic and electrochemical properties with strong potential for energy storage and supply applications (superconductivity, battery, thermoelectricity, hydrogen affinity, etc.) [22–26].

Although Mo_6_ cluster-based compounds were reported in the literature a long time ago and they have recently shown great potential for advanced applications [34–37], there are still large possibilities of improvement for further implementation into energy and environment applications. In order to fully exploit MCs, it is necessary to have a better understanding of the relations between chemical compositions, structural arrangements, and physical properties in MC-based compounds for a better selection of targeted properties and applications, as well as to develop scalable and industrially viable processes. For instance, some of us showed recently the effects of centro-symmetry in the Cs_2_Mo_6_Cl_14_.xH_2_O system on second harmonic generation [28]. On the other hand, we reported the design of transparent inorganic thin films based on octahedral Mo_6_ MCs deposited on indium tin oxide (ITO) glass by electrophoretic deposition (EPD) within a short time interval and at low cost [21]. Recently, multicomponent emission of [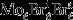]^2−^ cluster units has been reported [29]. Several excited triplet states resulting from complex de-excitation processes (breaking Kasha’s rule) have been evidenced. Quantum chemical studies suggest that the geometries of this excited triplet states are different from the ground state (S_0_) geometrical arrangement. These changes are either an elongation of one Mo-Mo bond or axial and non-axial elongations of one Mo apex. It turns out that absorption and emission properties of Mo_6_ cluster-based units are very sensitive to the nature of ligands and counter-ions, and more generally to matrix effects [9,30]. One of the goals of this work is to evidence if such a de-excitation cascade is also effective for another source of excitation (e-beam). Secondly, we aim at evaluating the luminescence efficiency over time upon highly energetic photon/electron irradiation (ageing), a key point to consider prior to effective applications such as energy conversion or field emission display (FED) applications [31]. To the best of our knowledge, no study of ageing of Mo_6_ cluster units upon different excitation sources has been reported to date. This work reports (i) the cathodoluminescence (CL) properties of representative face-capped [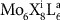]^2−^ cluster unit-based compounds; (ii) systematic and quantitative comparisons of photoluminescence (PL) properties; (iii) the influence of the excitation wavelength on their quantum yields (QY), defined as the ratio of the number of absorbed exciting photons and the number of emitted photons; and (iv) their luminescence stability over irradiation time.

## 2. Experimental section

### Description of the studied 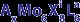 series

2.1.

We have synthesized and investigated seven compounds in the 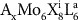 series – namely (Cs)_2_[Mo_6_Cl_14_] (**1**), (Cs)_2_[Mo_6_Br_14_] (**2**), (Cs)_2_[Mo_6_I_14_] (**3**), (Cs)_2_[Mo_6_I_8_(OOCC_2_F_5_)_6_] (**4**), ((n-C_4_H_9_)_4_N)_2_[Mo_6_Br_14_] (**5**), (NH_4_)_2_[Mo_6_Br_14_] (**6**), and (NH_4_)_2_[Mo_6_Br_8_(NCS)_6_] (**7**), according to the procedures reported in the literature, and whose structural view can be viewed in Supporting Figure 1 [27,32,33]. Those systems have been chosen since (i) **1**, **2**, and **3** constitute a ternary series of MCs solid-state compounds for which only the halogen atoms differ (Cl, Br, I); (ii) in the **2**, **5**, and **6** series, the [Mo_6_Br_14_]^2−^ cluster unit is kept unchanged, allowing study of the effect of counter-cations (Cs^+^, (n-C_4_H_9_)_4_N^+^ = TBA^+^, 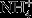); (iii) **1**, **4**, and **7** are the most emissive systems of the series. It may be noted that **4** and **7** contain an organic apical ligand (C_2_F_5_COO^−^) and SCN^−^, respectively, for which stability could be an issue. Actually, a continuous irradiation could affect the Mo-O and Mo-N bonds, respectively. Recently a lot of work has been devoted to the series of general formula 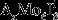
(OOCC_n_F_2n+1_)^a^_6_ (A = Cs^+^, (n-C_4_H_9_)_4_N^+^; *n* = 1, 2, and 3) owing to their remarkable luminescent properties. Among all face-capped clusters, the last-mentioned series exhibits the highest quantum yields [18,30,37–44].

**Figure 1.**
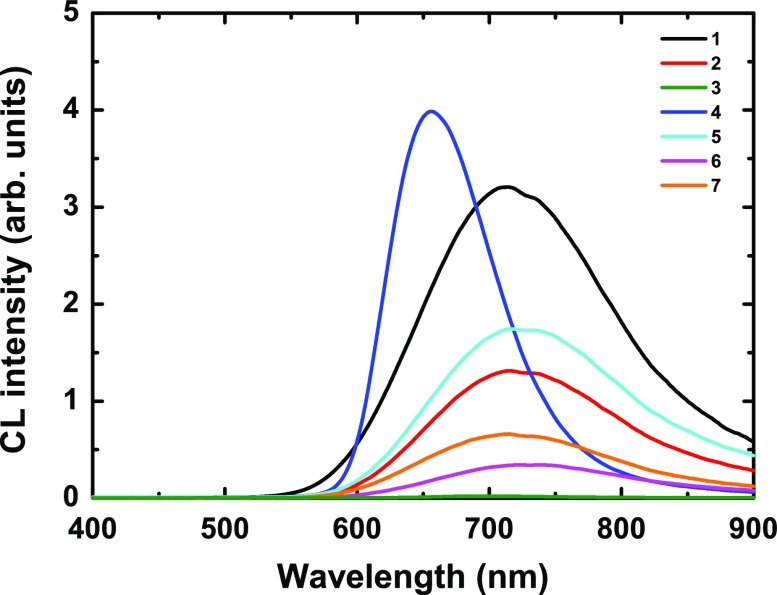
CL spectra of **1–7** compound series.

### 2.2. Description of the measurement techniques

The chemical composition and purity of all the compounds were monitored by X-ray diffraction (XRD) and scanning electron microscopy combined with energy-dispersive electron spectroscopy (SEM-EDS). QY measurements were made upon continuous irradiation of a 150 W Xe lamp, corresponding to a power density of ca. 0.5 μW cm^−2^, in standard atmosphere conditions using a system combining two-multichannel photodetectors (MCPD-9800: Otsuka Electronics) and a 3.3 inch integrating semi-sphere. Excitation wavelength was selected by a monochromator with an accuracy of ca. 3 nm. PL spectra were generated by combining UV–visible (250–800 nm) and IR data (800–1100 nm). The spectral resolution was 0.3 and 3 nm for UV–visible and IR, respectively. Time-dependent photo-irradiation was performed using a micro-PL system (HORIBA LabRam HR) equipped with fixed 325 nm He-Cd laser system (average source power of 20 mW and around 10 mW on the sample), corresponding to a power density of ca. 1000 mW cm^−2^, and a Perce type charge-coupled device (CCD) to investigate the PL evolution under photon excitation at ambient conditions. CL measurements were performed in a field emission SEM (Hitachi SU6600) equipped with a CL system (HORIBA MP32) at room temperature and 3 × 10^−3^ Pa. For CL measurements, a few milligrams of powders were deposited and hand-pressed on carbon tape for each composition. CL spectra were obtained at room temperature by a CCD (2048 channels, HORIBA Jobin-Yvon, Spectrum One), while CL mapping was carried out with a photomultiplier (Hamamatsu, R943–02). The applied voltage and beam current were set to 10 kV and 100 pA. The probe size was 12 nm, and the spectral resolution was about 0.2 nm.

## 3. Results and discussion

### 3.1. Cathodoluminescence properties of 1–7

The CL spectra of **1–7** are gathered in Figure 1. Except **3**, all compounds exhibit CL properties on a large spectral window ranging from 550 nm to more than 900 nm. The all-iodide compound **3** that hardly shows CL will not be subsequently considered. We suspect that **3** decomposes under e-beam irradiation similarly as under UV irradiation. Except for **4**, the CL spectra consist of an asymmetrical broad band with a maximum centered at around λ_max_ = 720 nm. CL spectrum of **4** shows a narrower blue-shifted band than that of the other members of the series (λ_max_ = 655 nm). For all the emission spectra except **4**, a small depletion is observed around 725 nm.

### 3.2. Photoluminescence properties of 1–7 and quantum yield evolutions

**Figure 2.**
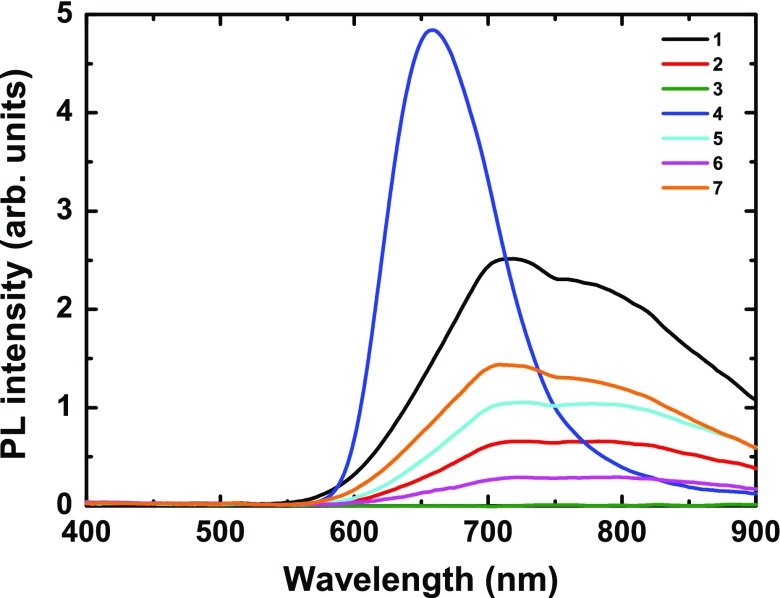
PL spectra of **1–7** compound series (λ_exc_ = 325 nm).

PL spectra of **1–7** upon 325 nm excitation generated by a Xe lamp are shown in Figure 2. For **1**, **2**, **5**, **6**, and **7**, the spectral PL shape consists of an asymmetric structured broad band centered roughly at λ_max_ = 715 nm. It is worth noting that this shape is more asymmetric than that found for CL. One can note that spectra of **2**, **5**, and **6** are almost flat between 715 and 790 nm. Contrary to the other samples, the PL spectrum of **4** consists of a less asymmetric broad band centered at λ_max_ = 655 nm, which is similar to that found in CL. As there is no observable PL emission for **3**, it will not be discussed further. It may also be noted that, for all the clusters except **4**, there is an inflexion point around 750 nm that may be related to self-absorption, as quantum chemical calculations performed on [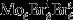]^2−^ relaxed excited states show absorption transitions in the NIR region (∼600, 750, 900 nm) [29]. It may be noted that the position of the inflexion point found for PL (750 nm) is different compared to that found for CL (725 nm). The full understanding of the origin of this position shift, probably related to the absorption of several excited triplet states, will require further combined and theoretical investigations.

**Figure 3.**
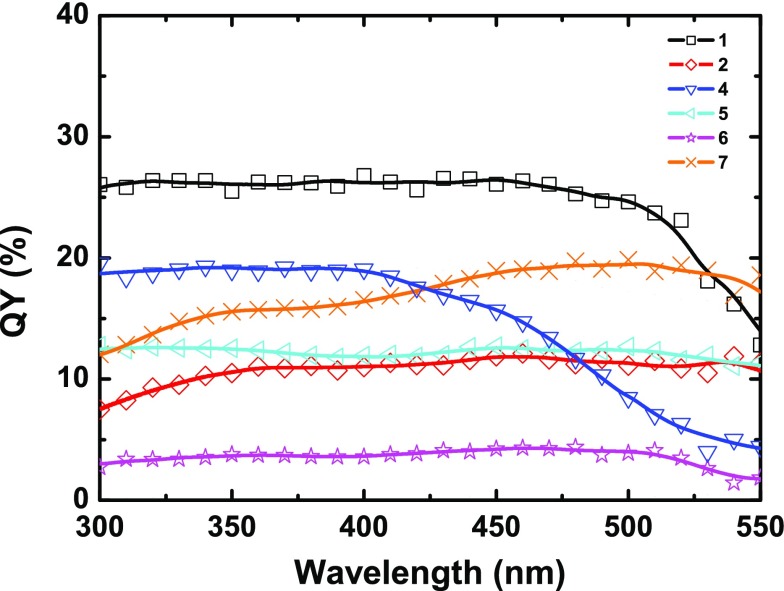
QY of **1–2** and **4–7** vs. excitation wavelength (300–550 nm).

Figure 3 shows the QY excitation wavelength dependence (λ_exc_ = 300–550 nm) of **1**, **2**, and **4–7**. The analysis of the curves reveals two behaviors: (i) for **1**, **4**, **5**, and **6**, a plateau with the maximum QY value is observed followed by a loss of efficiency as the excitation wavelength increases; (ii) for **2** and **7**, a smooth increase of QY is observed from 300 nm to roughly 550 nm. These different behaviors evidence that the emission strongly depends on the environment of the metallic Mo_6_ clusters: on one hand, the effect of X^i^ inner and L^a^ apical ligands forming the [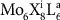]^2−^ unit, and, on the other hand, the effect of counter-cations [45]. It is worth noting that for **2**, **5**, and **6** that are based on the same [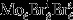]^2−^cluster unit, but differing in the nature of counter-cations, the two behaviors are found. This unambiguously proves that there exists a significant effect of the cations in the de-excitation processes.

Here we report the evolution of QY for several members of the 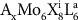 series as a function of the excitation wavelength. The measurements were carried out using the same experimental set-up and the same experimental conditions. 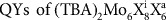 series were reported in previous works using a single excitation wavelength. In acetonitrile solutions, QYs of (TBA)_2_[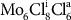] and (TBA)_2_[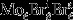] were reported by Maverick et al. to be 19% and 23% upon excitation at 436 nm [46]. Absolute quantum yields were estimated by comparison with 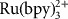 solutions. On the other hand, Kirakci et al. reported, by a comparative method with cresyl violet as reference upon excitation at 440 nm, that the QYs of (TBA)_2_[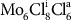], (TBA)_2_[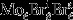], and (TBA)_2_[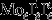] in acetonitrile solution are 15%, 13%, and 12%, respectively [30]. More recently, the QY of (TBA)_2_[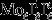] measured on powder upon excitation at 400 nm was reported to be 10% [45]. It is worth noting that the QY of parent compound (4-ViBnNMe_3_)_2_[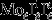] (4-ViBnNMe_3_ = trimethyl(4-vinylbenzyl)ammonium) measured on powders by the same authors and in the same conditions as (TBA)_2_[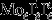] is only 1%. In the present study, QY could not be measured for Cs_2_[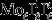]. For iodide clusters, those previous measurements showed that counter-cations play a significant role in luminescent properties. Actually, changes in the luminescent properties observed for the bromine series **2**, **5**, and **6** are related to the nature of the counter-cations. Counter-cations drive the packing and stacking of [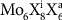]^2−^ clusters units within the crystal, and we suspect that a dense packing favors reabsorption phenomena. Additionally to optical reabsorption, the strong absorbance of **3** probably induces decomposition by overheating upon continuous irradiation. Luminescence and QY of **3** could thus not be measured. The substitution of iodide apical ligands by organic substituents substantially decreases these phenomena. QYs of powder samples of Cs_2_[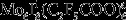] and its derivative (TBA)_2_[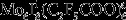] have been recently determined at 35% and 32%, respectively, by absolute photoluminescence QY measurement upon 380 nm excitation [44,47].

It has to be pointed out that except for **4**, which incorporates an organic ligand, the rest of the series shows the maximum QY in a large range of excitation energy from UV to the lower-energy green-region. This excitation window combined with NIR emission properties makes Mo_6_ clusters relevant down-conversion phosphorescent dyes for the design of solar cell concentrators for photovoltaic applications as already demonstrated by Lunt et al. for a series of [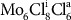]^2−^ based compounds [11,12]. Mo_6_ cluster compounds constitute an alternative to lead-based perovskites. Both families of compounds exhibit many analogies. It is worth mentioning that halogen ligands and cations play a significant role in optical properties of lead-based perovskites as demonstrated here for the 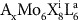 series [48,49].

### 3.3. Comparative analysis of CL and PL results

As stressed above, except for **4**, the spectral shape of the luminescence (PL and CL) deviates from the Gaussian function. This deviation is more pronounced for PL spectra. This is most probably related to the multicomponent nature of the emission. In order to quantify this matter, CL and PL spectra were normalized, and were then fitted with two Gaussian functions whose characteristics – maximum (E_max1_, E_max2_), full width at half maxima (FWHM_1_, FWHM_2_), and relative contributions (cont_1_, cont_2_) – are given in Table 1.

**Table 1.** Parameters of the Gaussian functions used to fit CL and PL spectra of **1**, **2**, and **4–7**: E_max1_, E_max2_, FWHM_1_, FWHM_2_ in eV; cont_1_ and cont_2_ percentages; coefficient of determination *R* ^2^. Values in italics were fixed (see text).

	E-beam excitation								Optical excitation						
	E_max1_	E_max2_	FWHM_1_	FWHM_2_	cont_1_	cont_2_	*R*^2^		E_max1_	E_max2_	FWHM_1_	FWHM_2_	cont_1_	cont_2_	*R*^2^
1	1.396	1.740	0.097	0.349	1.5	98.5	0.9998		1.448	*1.740*	0.245	0.316	31.4	68.6	0.9983
2	1.401	1.721	0.128	0.333	3.3	96.7	0.9997		1.463	*1.721*	0.298	0.302	41.1	58.9	0.9958
4	1.796	1.909	0.240	0.174	44.4	55.6	0.9988		*1.796*	*1.909*	0.222	0.170	47.3	52.7	0.9971
5	1.400	1.716	0.126	0.339	3.4	96.6	0.9996		1.447	*1.716*	0.270	0.311	34.7	65.3	0.9962
6	1.394	1.704	0.099	0.333	2.3	97.7	0.9998		1.447	*1.704*	0.245	0.310	32.5	67.5	0.9908
7	1.398	1.736	0.112	0.342	2.1	97.9	0.9998		1.463	*1.736*	0.258	0.316	27.4	72.6	0.9954

Except for **4**, all CL spectra are well fitted using a first Gaussian component centered at E_max1_ ~ 1.40 ± 0.01 eV with a FWHM of ~ 0.11 ± 0.02 eV and a second one at E_max2_ ~ 1.72 ± 0.02 eV with a FWHM of ~ 0.34 ± 0.01 eV, respectively. The first contribution is minor, representing less than 4% of the total spectra. A deeper analysis can be made taking into account the nature of the ligands and counter-cations. For compounds **2**, **5**, and **6**, which contain the same cluster unit [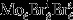]^2−^, the energies and bandwidths of the two components are relatively similar with a maximum deviation of less than 0.02 eV. Changing the apical ligands from bromines (**6**) to thiocyanates (**7**) leads to a slight shift of E_max2_ energy (1.704 to 1.736 eV). Changing the bromine apical and inner ligands in **2** by chlorine in **1** barely modifies the emission properties from the energetic point of view. Such behavior has been reported for the PL of 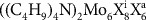 (X = Cl, Br) [46]. Overall, one can conclude that the nature of the ligands and of the counter-cations for this group (**1**, **2**, **5–7**) plays a limited role on the energies of CL emissions, but, as previously highlighted, it has more noticeable effects on their emission intensities.

The CL properties of **4** are different from the rest of the series described above. One can note that: (i) the maximum of emission is blue-shifted; (ii) the emission intensity is the highest of the whole series; (iii) the two components are close in energy (E_max1_ = 1.796 eV, E_max2_ = 1.909 eV); and (iv) the first contribution reaches 44.4%, which strongly differs from other compounds for which the first component is minor. The substitution of apical iodides by pentafluoropropionates (C_2_F_5_COO^−^) going from **3** to **4** induces thus an incredible enhancement of emissive properties along with a strong blue-shift compared to the other member of the series. From this investigation and comparison between **4** and the other member of the series, it appears that, for the same [Mo_6_X^i^_8_]^4+^ cluster core, CL characteristics are more sensitive to the nature of apical ligands, in particular changing inorganic L^a^ ligands by organic pentafluoropropionate group, than to that of the counter-cations. This can be understood taking into account the molecular character of the [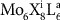]^2−^ unit. Frontier molecular orbitals (MO) of the cluster unit (singlet ground state S_0_) depend on the nature of ligands [50–52]. Consequently, the triplet (T_1+n_) and singlet (S_1+n_) excited states are also strongly affected by the nature of the apical ligands since they formally result from S_0_ electronic MO transitions. On the other hand, counter-cations mainly affect the crystal packing and, to a much lower extent, the ionic character of the Mo-L^a^ bond and the excited-state geometry relaxations. Judicious choice of apical ligands would enable emissive properties of Mo_6_-based compounds to be enhanced and optimized.

CL fitting procedure was applied to the PL spectra. While in PL, one incident photon generates usually one electron-hole pair, in CL, one incident electron generates thousands of pairs. This leads to significant differences in the excited-state populations between optical and e-beam excitation, and results in different spectral shapes due to different non-radiative energy losses occurring before emission. Taking into account lifetime of the excited states, re-absorption can also occur [29]. As it can be seen in Supporting Figure 2, there is a good match between the CL and PL spectra in the high-energy region (>1.68 eV). This part of the emission corresponds to the second component of the CL spectra, which is very accurately defined since it covers 96% of CL (except for **4**). For this reason, PL fits were performed by fixing E_max2_ to the value obtained after CL refinements. The values of Ε_max1_, Ε_max2_, FWHM_1_, and FWHM_2_ and relative contributions (cont_1_, cont_2_) of the two Gaussian components are given in Table 1. It turns out that the present results for (Cs)_2_[Mo_6_Br_14_] (**2**) and ((n-C_4_H_9_)_4_N)_2_[Mo_6_Br_14_] (**5**) are in full agreement with previously reported PL steady state measurements, despite the fact that the experimental set-up and excitation sources are different [29]. In this previous work, the doublet nature of the emission of [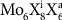]^2−^ in **2** and **5** metal cluster units has been demonstrated by a combined theoretical, steady-state and time-resolved PL investigation. Thus, from the PL excitation maps, the energies of the emission maxima (for λ_exc_ = 380 nm) of the two contributive emissions could be extracted as E_max1_ = 1.463 eV and E_max2_ = 1.721 eV (845 and 719 nm, respectively), and E_max1_ = 1.447 eV and E_max2_ = 1.716 eV (856 nm and 722 nm, respectively) for **2** and **5**, respectively. The corresponding ratios between the first and second contributions (cont_1_/cont_2_) are ~41/59 for **2** and ~35/65 for **5**. This set of convergent results proves the doublet structure of luminescence for two [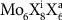]^2−^ cluster unit-based compounds differing by the counter-cation nature. The same behavior is suspected for the rest of the series. For the third compound of the all-bromide series, **6**, the maxima of energy Ε_max1_ and Ε_max2_ are the same as those of **2** and **5**. The ratio between each contribution is the same as that of **5** (~33/67 compared to ~35/65), revealing a similar impact of the counter-cation environment. This result is coherent with the fact that both **5** and **6** are ammonium-type cations ((n-C_4_H_9_)_4_N^+^ for **5**; 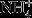 for **6**). Going from **6** to **7**, i.e. replacing apical bromine atoms by thiocyano groups, a blue-shift of both Ε_max1_ and Ε_max2_ is observed along with a decrease of cont_1_/cont_2_ ratio from ~33/67 to ~27/73. For the whole series, both components show rather similar FWHM, which are mainly due to vibrational phenomena (from 0.17 to 0.32 eV).

Similarly to CL, the PL properties of **3** and **4** are very different from those of the others members of the series. While **3** hardly emits, **4** exhibits a strong blue-shifted luminescence compared to the other member of the series. Moreover, since the PL and CL spectral shapes for **4** are very similar, both E_max1_ and E_max2_ were fixed to the values found in CL for PL fittings. The slight difference between PL and CL spectra for **4** is due to a very slight evolution of cont_1_/cont_2_ ratios (~47/53 for PL compared to ~44/56 found for CL). It suggests that, for **4**, both types of excitation (photon and electron-beam) lead to the same emissive triplet states and thus most probably to similar non-radiative de-excitation pathways. The substitution of apical iodide by C_2_F_5_COO^−^ going from **3** to **4** affords a modified [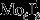
(OOCC_2_F_5_)]^2−^ cluster unit with outstanding enhancement of emissive properties along with a strong blue-shift compared to the other members of the series.

### 3.4. Luminescence intensity decay upon continuous laser optical irradiation

**Figure 4.**
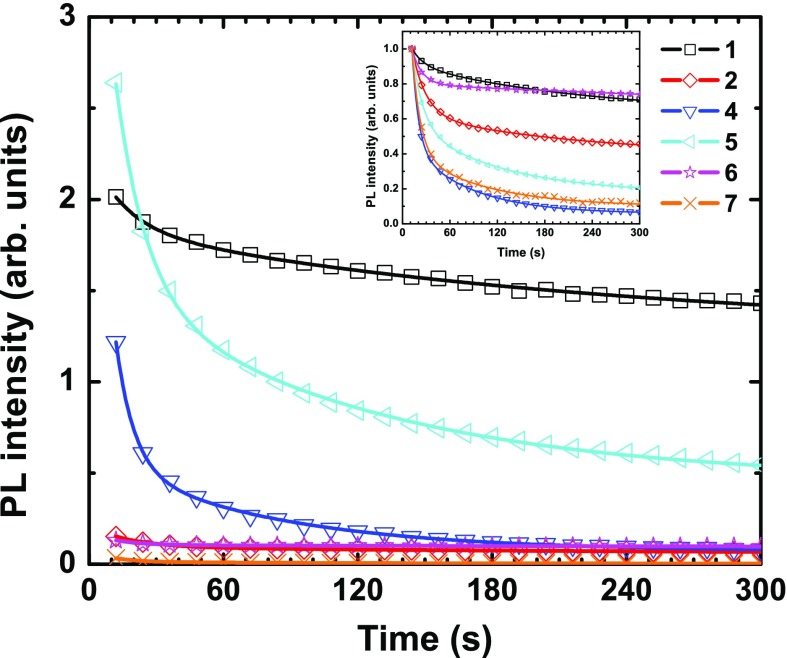
PL intensity decay at maximum of emission of **1**, **2**, and **4–7** upon continuous 325 nm laser irradiation (average source power of 20 mW – around 10 mW on the sample). Inset: normalized curves.

The evolution in time of the PL intensity of the series of compounds under study upon high-power continuous laser irradiation at a wavelength of 325 nm is given in Figure 4. The experiment was performed over 300 s. Normalized curves are given as inset. The curves can be fitted with a double exponential: A_1_ 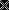 exp(−x/τ_1_) + A_2_ 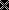 exp(−x/τ_2_) + y_0_, whose A_1/2_ and τ_1/2_ are reported in Table 2. At this stage, it has to be pointed out that while no noticeable change in intensity emission over the time was observed during the QY measurements, which last roughly 10 min, important decreases of intensity are observed for all compounds in the present experiment. It may be related to a difference in light intensity, which was lower for QY measurements (Xe lamp). As previously reported, the absorption properties and notably the absorbance at 325 nm depend on the composition of each studied system. It means that the structural damage or decomposition phenomena originating from heat generation upon high-power irradiation differs from one composition to another [13,53,54]. In the halogenide series **1**, **2**, and **3**, the heavier the halogen is, the higher its UV–visible absorbance is [55]. Compound **3** exhibits the strongest absorbance, and it appears to be the worst emitter. This result suggests that important non-radiative processes occur after excitation, self-reabsorption, or decomposition phenomena. For other cluster compositions, QY can be high with a weak absorbance leading to inferior luminescence properties.

**Table 2.** Fitting parameters by a double exponential of PL (Figure 4) and CL (Figure 5) intensity decays. The loss percentage after 300s irradiation and the QY at 325 nm (QY325nm in percentage) extracted from Figure 3 are also reported.

	A_1_	Optical excitation						A_1_	E-beam excitation			
		τ_1_ (s)	A_2_	τ_2_ (s)	Loss (%)	QY_325 nm_ (%)			τ_1_ (s)	A_2_	τ_2_ (s)	Loss (%)
1	0.42	15	0.58	226	29	26.3		3.03	13	1.67	87	90.2
2	0.11	17	0.04	207	45	9.4		0.75	24	0.62	144	76.6
4	3.05	8.1	0.53	76	94	19.3		34.5	4.2	1.21	28	99.5
5	2.81	13	1.13	114	79	12.6		2.00	6.6	1.14	210	71.6
6	0.07	13	0.04	1300	26	3.4		0.34	9.6	0.20	135	82.7
7	0.06	10	0.01	93	88	14.2		0.64	9.1	0.43	76	88.3

For the design of effective PL-based devices, good candidates should absorb strongly at the working excitation wavelengths, and have a high quantum yield resulting in high PL intensity (luminance). Ageing, e.g. loss of intensity, or change in energy of emission, is thus an element to consider for the selection of phosphorescent dyes. Laser irradiation can cause photo-induced phase transition and structural damages via local heat generation. Considering initial emission intensity, the best systems within the studied series are **1**, **4**, and **5** as shown in Figure 4. Additionally, if ageing is taken into account, the compound that is the most resistant to a strong and continuous photon irradiation is **1**, with still 70% of luminescence intensity after 300 s of laser irradiation. For the two other best candidates, **4** and **5**, the decay regime is very abrupt since more than 50% of their intensity is lost after only 60 s. The compounds **1** and **5** are the only ones for which the emission is still significant after 300 s. This conclusion could be different for another wavelength of irradiation since all processes (excitation and de-excitation) are wavelength-dependent (absorption, non-radiative decay, emissive and non-emissive excited-state population).

### 3.5. Luminescence intensity decay upon continuous e-beam irradiation

**Figure 5.**
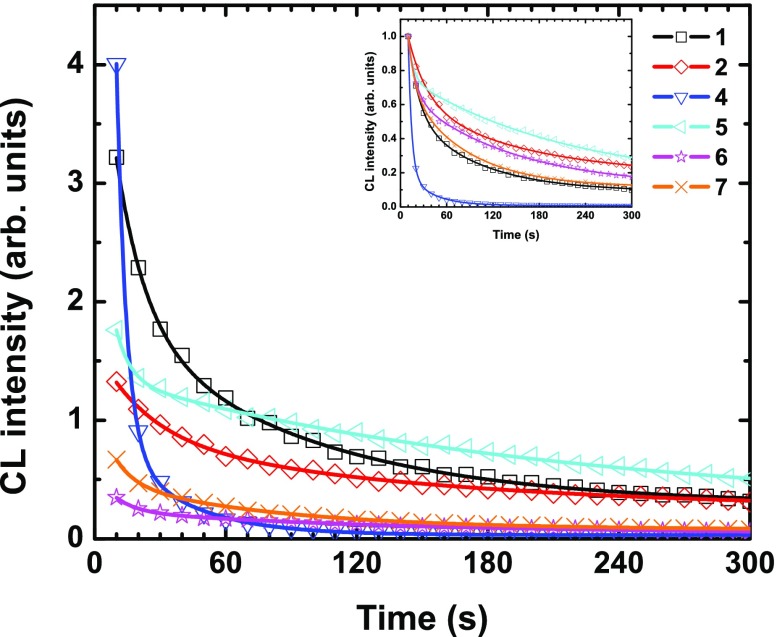
CL intensity decay at maximum of emission of **1**, **2**, and **4–7** upon electron irradiation. Inset: normalized curves.

Ageing of a luminescent material can also be evaluated using e-beam excitation. Electron-induced damages can indifferently affect an organic or inorganic sample through electrostatic charging, heating, ionization damage (radiolysis), displacement damage, sputtering, or hydrocarbon contamination [56,57]. Luminescence intensity decay was studied for the whole series (Figure 5). CL decay characteristics are given in Table 2 together with those of PL (corresponding to Figure 4).

As shown in Figure 5, when a continuous e-beam is applied, one can observe a drastic decrease in intensity. Two decay regimes are found. For **4**, an abrupt loss of intensity is found, leading to a total extinction of luminescence after roughly 120 s. For the rest of the series, a smoother decrease is measured. Only the [Mo_6_Br_14_]^2−^ cluster unit-based compounds (**2**, **5**, and **6**) are still emitting at more than 40% of their initial intensity after 60 s of irradiation. After 300 s, the remaining intensity ranges from 10% to 30% of the initial intensity. Taking into account the absolute intensity, the most emissive compound after 300 s is **5** followed by **1** and **2**. For the rest of the series, no significant CL is measured. These results suggest that for a targeted application, an 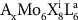 compound could be tailor-made by a judicious choice of ligands and counter-cations. For applications requiring low-power photo-irradiation, **4** would give the highest emission intensity, while for those needing high-power or long-term photo-irradiation, **1** seems to be more relevant. Compounds **2** and **5** may be a better choice for FED applications.

## 4. Conclusions

The luminescence behaviors of seven representative members of the 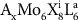 series were investigated in order to evaluate the influence of the nature of ligands bound to the Mo_6_ cluster, as well as the nature of counter-cations on QY, PL, CL, and ageing upon continuous light source and a continuous electron beam irradiation.

The PL multicomponent emission previously found for Mo_6_ cluster-based bromides (Cs)_2_[Mo_6_Br_14_] (**2**) and ((n-C_4_H_9_)_4_N)_2_[Mo_6_Br_14_] (**5**) has been enlarged to other members of 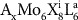. Moreover, while two peaks are observed in PL, the high-energy emission component is predominant in CL with the exception of (Cs)_2_[Mo_6_I_8_(OOCC_2_F_5_)_6_] (**4**), which exhibits a CL doublet. The latter shows blue-shifted emission spectra whatever the nature of the excitation source (photon or electron), revealing that the same emissive triplet states are populated. The intensity of luminescence of **4** is one of the most important among the series, but it decreases dramatically over time. It turns out that (Cs)_2_[Mo_6_Cl_14_] (**1**), **2**, and **5** are the most robust systems upon irradiation.

Additionally, understanding ageing mechanisms is mandatory to improve the luminescence stability of the MCs and thus enlarge their potential application fields. For that purpose, predictive molecular engineering, combining theoretical and experimental approaches, may greatly contribute in the fine-tuning of the luminescence properties of cluster-based compounds [28,50,51]. Another strategy lies in materials engineering consisting in embedding MCs into a mediating (active) matrix. This should enhance the stability of the luminescence properties of the MCs by favoring heat dissipation and limiting non-radiative processes for long-term applications [10,47,58,59].

## Disclosure statement

No potential conflict of interest was reported by the authors.

## Funding

The study was financially supported by Saint-Gobain (France), CNRS, University of Rennes 1, and NIMS through the Laboratory for Innovative Key Materials and Structures (LINK UMI 3629). M.A.C. thanks ANR Clustomesogens ANR-13-BS07-0003-01.

## Supplemental data

The supplemental material for this paper is available online at https://doi.org/10.1080/14686996.2017.1338496.

## Supplementary Material

Suppl.zip

## References

[1] Lewis NS, Nocera DG Powering the planet: chemical challenges in solar energy utilization. Proc Natl Acad Sci. 2006;103(43):15729–15735. 10.1073/pnas.0603395103 17043226 PMC1635072

[2] Ariga K, Hill JP, Ji Q Layer-by-layer assembly as a versatile bottom-up nanofabrication technique for exploratory research and realistic application. Phys Chem Chem Phys. 2007;9:2319–2340. 10.1039/b700410a 17492095

[3] Lu Y, Chen W Sub-nanometre sized metal clusters: from synthetic challenges to the unique property discoveries. Chem Soc Rev. 2012;41:3594–3623. 10.1039/c2cs15325d 22441327

[4] Ghosh B, Shirahata N Colloidal silicon quantum dots: synthesis and luminescence tuning from the near-UV to the near-IR range. Sci Technol Adv Mater. 2014;15(1):014207–14214. 10.1088/1468-6996/15/1/014207 27877634 PMC5090595

[5] Sun HT, Sakka Y Luminescent metal nanoclusters: controlled synthesis and functional applications. Sci Technol Adv Mater. 2014;15(1):014205–014213. 10.1088/1468-6996/15/1/014205 27877632 PMC5090593

[6] Cordier S, Grasset F, Molard Y, et al Inorganic molybdenum octahedral nanosized cluster units, versatile functional building block for nanoarchitectonics. J Inorg Organomet Polym. 2015;25(2):189–204. 10.1007/s10904-014-0112-2

[7] Perrin A, Perrin C The molybdenum and rhenium octahedral cluster chalcohalides in solid state chemistry: from condensed to discrete cluster units. C R Chim. 2012;15(9):815–836. 10.1016/j.crci.2012.07.004

[8] Fedorov V As they were born in Siberia. J Clust Sci. 2015;26(1):3–15

[9] Kuttipillai PS, Zhao Y, Traverse CJ, et al Phosphorescent nanocluster light-emitting diodes. Adv Mater. 2016;28(2):320–326. 10.1002/adma.201504548 26568044

[10] Molard Y Liquid crystalline hybrid nanomaterials containing functional metal nanoclusters. Acc Chem Res. 2016;49:1514–1523. 27434708 10.1021/acs.accounts.6b00236

[11] Lunt RR, Kuttipillai PS, inventor Nanocluster based light emitting device. United States patent US 2015/0069366 A1 2015 Mar 12.

[12] Zhao Y, Lunt RR Transparent luminescent solar concentrators for large-area solar windows enabled by massive stokes-shift nanocluster phosphors. Adv Energy Mater. 2013;3(9):1143–1148. 10.1002/aenm.v3.9

[13] Zhao Y, Meek GA, Levine BG, et al Near-infrared harvesting transparent luminescent solar concentrators. Adv Opt Mater. 2014;2(7):606–611. 10.1002/adom.201400103

[14] Renaud A, Grasset F, Dierre B, et al Inorganic molybdenum clusters as light-harvester in all inorganic solar cells: a proof of concept. ChemistrySelect. 2016;1(10):2284–2289. 10.1002/slct.201600508

[15] Aubert T, Cabello-Hurtado F, Esnault MA, et al Extended investigations on luminescent Cs_2_[Mo_6_Br _14_]@SiO_2_ nanoparticles: physico-structural characterizations and toxicity studies. J Phys Chem C. 2013;117(39):20154–20163. 10.1021/jp405836q

[16] Kirakci K, Kubát P, Fejfarová K, et al X-ray inducible luminescence and singlet oxygen sensitization by an octahedral molybdenum cluster compound: a new class of nanoscintillators. Inorg Chem. 2016;55(2):803–809. 10.1021/acs.inorgchem.5b02282 26702498

[17] Solovieva AO, Vorotnikov YA, Trifonova KE, et al Cellular internalisation, bioimaging and dark and photodynamic cytotoxicity of silica nanoparticles doped by {Mo_6_I_8_}^4+^ metal clusters. J Mater Chem B. 2016;4:4839–4846. 10.1039/C6TB00723F 32263142

[18] Neaime C, Amela-Cortes M, Grasset F, et al Time-gated luminescence bioimaging with new luminescent nanocolloids based on [Mo_6_I_8_(C_2_F_5_COO)_6_]^2−^ metal atom clusters. Phys Chem Chem Phys. 2016;18:30166–30173. 10.1039/C6CP05290H 27778003

[19] Beltrán A, Mikhailov M, Sokolov MN, et al A photobleaching resistant polymer supported hexanuclear molybdenum iodide cluster for photocatalytic oxygenations and photodynamic inactivation of Staphylococcus aureus. J Mater Chem B. 2016;4:5975–5979. 10.1039/C6TB01966H 32263486

[20] Truong TG, Dierre B, Grasset F, et al Visible tunable lighting system based on polymer composites embedding ZnO and metallic clusters: from colloids to thin films. Sci Technol Adv Mater. 2016;17(1):443–453. 10.1080/14686996.2016.1202724 27877895 PMC5101959

[21] Nguyen TKN, Grasset F, Dierre B, et al Fabrication of transparent thin film of octahedral molybdenum metal clusters by electrophoretic deposition. ECS J Solid State Sci Technol. 2016;5(10):R178–R186. 10.1149/2.0291610jss

[22] Chevrel R, Sergent M, Prigent J. Sur de nouvelles phases sulfurées ternaires du molybdène [On novel ternary molybdenum sulfide phases]. J Solid State Chem. 1971;3:515–519 French.

[23] Aurbach D, Lu Z, Schechter A, et al Prototype systems for rechargeable magnesium batteries. Nature. 2000;407:724–727. 10.1038/35037553 11048714

[24] Aurbach D, Suresh SG, Levi E, et al Progress in rechargeable magnesium battery technology. Adv Mater. 2007;19(23):4260–4267. 10.1002/(ISSN)1521-4095

[25] Gougeon P, Gall P, Al Rahal Al Orabi R, et al Synthesis, crystal and electronic structures, and thermoelectric properties of the novel cluster compound Ag_3_In_2_Mo_15_Se_19_ . Chem Mater. 2012;24(15):2899–2908. 10.1021/cm3009557

[26] Fujii S, Horiguchi T, Akagi S, et al Quasi-one-step six-electron electrochemical reduction of an octahedral hexanuclear molybdenum(II) cluster. Inorg Chem. 2016;55(20):10259–10266. 10.1021/acs.inorgchem.6b01525 27685662

[27] Potel M, Perrin C, Perrin A, et al New families of ternary molybdenum (II) chlorides with octahedral Mo_6_ clusters. Mat Res Bull. 1986;21(10):1239–1245. 10.1016/0025-5408(86)90053-X

[28] Saito N, Wada Y, Lemoine P, et al Theoretical and experimental determination of the crystal structures of cesium–molybdenum chloride. Jpn J Appl Phys. 2016;55:075502 10.7567/JJAP.55.075502

[29] Costuas K, Garreau A, Bulou A, et al Combined theoretical and time-resolved photoluminescence investigations of [Mo_6_Br^i^ _8_Br^a^ _6_]^2−^ metal cluster units: evidence of dual emission. Phys Chem Chem Phys. 2015;17:28574–28585. 10.1039/C5CP03960F 26435303

[30] Kirakci K, Kubát P, Langmaier J, et al A comparative study of the redox and excited state properties of (*n*Bu_4_N)_2_[Mo_6_X_14_] and (*n*Bu_4_N)_2_[Mo_6_X_8_(CF_3_COO)_6_] (X = Cl, Br, or I). Dalton Trans. 2013;42:7224–7232. 10.1039/c3dt32863e 23532319

[31] Dierre B, Yuan XL, Sekiguchi T Low-energy cathodoluminesence microscopy for the characterization of nanostructures. Sci Technol Adv Mater. 2010;11:043001 10.1088/1468-6996/11/4/043001 27877341 PMC5090332

[32] Kirakci K, Cordier S, Perrin C Synthesis and characterization of Cs_2_Mo_6_X_14_ (X = Br or I) hexamolybdenum cluster halides: efficient Mo6 cluster precursors for solution chemistry syntheses. Z Anorg Allg Chem. 2005;631(2-3):411–416. 10.1002/(ISSN)1521-3749

[33] Schäfer H, Schnering HGV, Tillack J, et al Neue Untersuchungen über die Chloride des Molybdäns. Z Anorg Allg Chem. 1967;353(5-6):281–310. 10.1002/(ISSN)1521-3749

[34] Sheldon JC Polynuclear complexes of molybdenum(II). Nature. 1959;184:1210–1213. 10.1038/1841210a0

[35] Sheldon JC Chloromolybdenum(II) compounds. J Chem Soc. 1960;1007–1014. 10.1039/jr9600001007

[36] Cotton FA, Curtis NF Some new derivatives of the octa-μ3-chlorohexamolybdate(II),[Mo_6_Cl_8_]^4+^, ion. Inorg Chem. 1965;4(2):241–244. 10.1021/ic50024a025

[37] Sokolov MN, Mikhailov MA, Peresypkina EV, et al Highly luminescent complexes [Mo_6_X_8_(n-C_3_F_7_COO)_6_]^2−^ (X = Br, I). Dalton Trans. 2011;40:6375–6377. 10.1039/c1dt10376h 21594270

[38] Kirakci K, Kubát P, Dušek M, et al A highly luminescent hexanuclear molybdenum cluster – A promising candidate toward photoactive materials. Eur J Inorg Chem. 2012;2012(19):3107–3111. 10.1002/ejic.v2012.19

[39] Sokolov MN, Mikhailov MA, Brylev KA, et al Alkynyl complexes of high-valence clusters. Synthesis and luminescence properties of [Mo_6_I_8_(C≡CC(O)OMe)_6_]^2-^, the first complex with exclusively organometallic outer ligands in the family of octahedral M_6_X_8_ clusters. Inorg Chem. 2013;52(21):12477–12481. 10.1021/ic401377g 24127646

[40] Efremova OA, Shestopalov MA, Chirtsova NA, et al A highly emissive inorganic hexamolybdenum cluster complex as a handy precursor for the preparation of new luminescent materials. Dalton Trans. 2014;43:6021–6025. 10.1039/c3dt53126k 24457554

[41] Kirakci K, Fejfarová K, Kučeráková M, et al Hexamolybdenum cluster complexes with pyrene and anthracene carboxylates: ultrabright red emitters with the antenna effect. Eur J Inorg Chem. 2014;14:2331–2336. 10.1002/ejic.201402076

[42] Kirakci K, Kubát P, Kučeráková M, et al Water-soluble octahedral molybdenum cluster compounds Na_2_[Mo_6_I_8_(N_3_)_6_] and Na_2_[Mo_6_I_8_(NCS)_6_]: syntheses, luminescence, and *in vitro* studies. Inorg Chim Acta. 2016;441:42–49. 10.1016/j.ica.2015.10.043

[43] Mikhailov MA, Brylev KA, Virovets AV, et al Complexes of Mo_6_I_8_ with nitrophenolates: synthesis and luminescence. New J Chem. 2016;40(2):1162–1168. 10.1039/C5NJ02246K

[44] Mikhailov MA, Brylev KA, Abramov PA, et al Synthetic tuning of redox, spectroscopic, and photophysical properties of {Mo_6_I_8_}^4+^ core cluster complexes by terminal carboxylate ligands. Inorg Chem. 2016;55(17):8437–8445. 10.1021/acs.inorgchem.6b01042 27505303

[45] Vorotnikov YA, Mikhailov MA, Brylev KA, et al Synthesis, crystal structure, and luminescence properties of complexes (4-ViBnNMe_3_)_2_[{M_6_(μ_3_-I)_8_}I_6_] (M = Mo, W; (4-ViBnNMe_3_)^+^ is trimethyl(4-vinylbenzyl)ammonium). Russ Chem Bull. 2015;64(11):2591–2596. 10.1007/s11172-015-1194-x

[46] Maverick AW, Najdzionek JS, MacKenzie D, et al Spectroscopic, electrochemical, and photochemical properties of molybdenum(II) and tungsten(II) halide clusters. J Am Chem Soc. 1983;105(7):1979–1882.

[47] Amela-Cortes M, Molard Y, Paofai S, et al Versatility of the ionic assembling method to design highly luminescent PMMA nanocomposites containing [M_6_Q^i^ _8_L^a^ _6_]^n-^ octahedral nano-building blocks. Dalton Trans. 2016;45:237–245. 10.1039/C5DT03734D 26599524

[48] Katan C, Pedesseau L, Kepenekian M, et al Interplay of spin–orbit coupling and lattice distortion in metal substituted 3D tri-chloride hybrid perovskites. J Mater Chem A. 2015;3:9232–9240. 10.1039/C4TA06418F

[49] Slavney AH, Smaha RW, Smith IC, et al Chemical approaches to addressing the instability and toxicity of lead−halide perovskite absorbers. Inorg Chem. 2017;56:46–55. 10.1021/acs.inorgchem.6b01336 27494338

[50] Ramirez-Tagle R, Arratia-Pérez R Electronic structure and molecular properties of the [Mo_6_X_8_L_6_]^2−^; X = Cl, Br, I; L = F, Cl, Br, I clusters. Chem Phys Lett. 2008;460(4–6):438–441. 10.1016/j.cplett.2008.06.035

[51] Ramirez-Tagle R, Arratia-Pérez R The luminescent [Mo_6_X_8_(NCS)_6_]^2−^ (X = Cl, Br, I) clusters: a computational study based on time-dependent density functional theory including spin–orbit and solvent-polarity effects. Chem Phys Lett. 2008;455(1-3):38–41. 10.1016/j.cplett.2008.02.037

[52] Efremova OA, Vorotniknov YA, Brylev KA, et al Octahedral molybdenum cluster complexes with aromatic sulfonate ligands. Dalton Trans. 2016;45:15427–15435. 10.1039/C6DT02863B 27605435

[53] El Mendili Y, Bardeau JF, Randrianantoandro N, et al Structural behavior of laser-irradiated γ-Fe_2_O_3_ nanocrystals dispersed in porous silica matrix: γ-Fe_2_O_3_ to α-Fe_2_O_3_ phase transition and formation of ε-Fe_2_O_3_ . Sci Technol Adv Mater. 2016;17(1):597–609. 10.1080/14686996.2016.1222494 27877906 PMC5101921

[54] Bertoni R, Lorenc M, Cailleau H, et al Elastically driven cooperative response of a molecular material impacted by a laser pulse. Nature Mater. 2016;15:606–610. 10.1038/nmat4606 27019383

[55] Grasset F, Dorson F, Cordier S, et al Water-in-oil microemulsion preparation and characterization of Cs_2_[Mo_6_X_14_]@SiO_2_ phosphor nanoparticles based on transition metal clusters (X = Cl, Br, and I). Adv Mat. 2008;20(1):143–148. 10.1002/(ISSN)1521-4095

[56] Egerton RF, Li P, Malac M Radiation damage in the TEM and SEM. Micron. 2004;35(6):399–409. 10.1016/j.micron.2004.02.003 15120123

[57] Nagatomi T, Nakamura H, Takai Y et al Approach to quantitative evaluation of electron-induced degradation of SiO_2_ film surface with different amounts of carbon contaminations. e-J Surf Sci Nanotech 2011;9:277–288. 10.1380/ejssnt.2011.277

[58] Efremova OA, Brylev KA, Vorotnikov YA, et al Photoluminescent materials based on PMMA and a highly-emissive octahedral molybdenum metal cluster complex. J Mat Chem C. 2016;4:497–503. 10.1039/C5TC03204K

[59] Uoyama H, Goushi K, Shizu K, et al Highly efficient organic light-emitting diodes from delayed fluorescence. Nature. 2012;492:234–238. 10.1038/nature11687 23235877

