# Supplementary figures and images for: Mo6 cluster-based compounds for energy conversion applications: comparative study of photoluminescence and cathodoluminescence
Source: Sci Technol Adv Mater. 2017 Jul 3;18(1):458–66. doi: 10.1080/14686996.2017.1338496 (PMC5508363; doi:10.1080/14686996.2017.1338496)

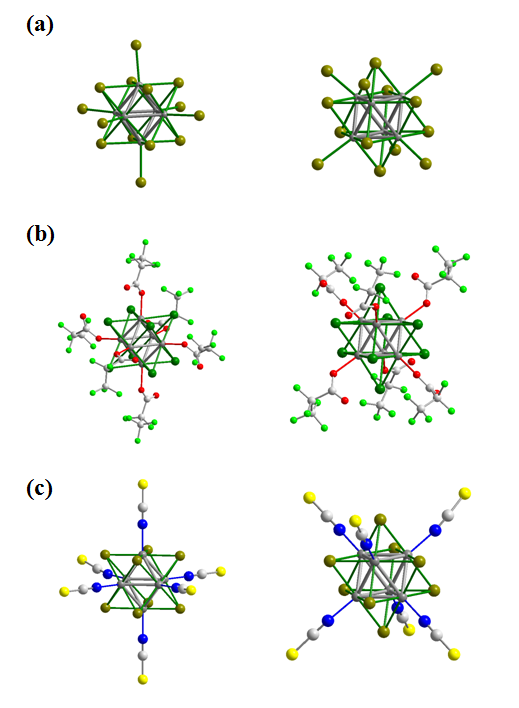

Supplement: Suppl.zip [file tsta_a_1338496_sm0883.zip › Figure SI1.bmp]
